# *Streptococcus mitis* and *Prevotella melaninogenica* Influence Gene Expression Changes in Oral Mucosal Lesions in Periodontitis Patients

**DOI:** 10.3390/pathogens12101194

**Published:** 2023-09-26

**Authors:** Uros Tomic, Nadja Nikolic, Jelena Carkic, Djordje Mihailovic, Drago Jelovac, Jelena Milasin, Ana Pucar

**Affiliations:** 1 Clinic for Periodontology and Oral Medicine, School of Dental Medicine, University of Belgrade, 11000 Belgrade, Serbia; anapucar@gmail.com; 2 Department of Human Genetics, School of Dental Medicine, University of Belgrade, 11000 Belgrade, Serbia; jelena.carkic@stomf.bg.ac.rs (J.C.); jelena.milasin@stomf.bg.ac.rs (J.M.); 3 Department of Dentistry, Faculty of Medical Sciences Pristina, University of Pristina, 38220 Kosovska Mitrovica, Serbia; drdjordjemihailovic@gmail.com; 4 Clinic for Maxillofacial Surgery, School of Dental Medicine, University of Belgrade, 11000 Belgrade, Serbia; jbdrago@gmail.com

**Keywords:** oral pathogenic bacteria, chronic inflammation, oral mucosal lesions, oral potentially malignant disorders, *Prevotella melaninogenica*, *Streptococcus mitis*

## Abstract

Oral microbiome disruptions in periodontitis are related to the chronic inflammatory reactions that could in turn lead to the development of multiple oral diseases. The objective of the study was to assess the frequencies of *Streptococcus mitis*, *Prevotella melaninogenica*, and *Prevotella intermedia* in oral benign lesions, oral potentially malignant disorders (OPMDs), and oral squamous cell carcinomas (OSCCs) and investigate the impact of these bacteria on the expression patterns of the selected (potential) target genes (*PI3CA*/*AKT2*/*mTOR*, *DUSP16*/*MAPK14*, and *COX2*). After sample collection (25 benign lesions, 30 OPMDs, and 35 OSCCs) and DNA/RNA extraction, quantitative real-time polymerase chain reaction (qPCR) was performed to detect bacterial presence and assess relative gene expression levels in different lesion groups. *Prevotella melaninogenica* was the most prevalent of the three analyzed bacteria, with the frequency being 60% in benign lesions, 87% in OPMDs (*p* = 0.024), and 77% in OSCC. The OPMD tissues in which *Prevotella melaninogenica* was present exhibited a higher expression level of *AKT2* (*p* = 0.042). Significantly lower expression of *DUSP16* was observed in OSCC tissues containing *Streptococcus mitis* (*p* = 0.011). The obtained results indicate a substantial contribution of *P. melaninogenica* and *Str. mitis* in the pathogenesis of oral mucosal lesions, possibly via *AKT2* upregulation and *DUSP16* downregulation.

## 1. Introduction

The oral cavity is a highly dynamic but at the same time a well-controlled microenvironment; its variations provide fertile ground for the development of numerous pathologies. One of the most important factors in maintaining oral homeostasis is the oral microbiome, which is the second most diverse microbial community in the human body, harboring over 700 bacterial species so far identified [1]. Pertubations in quantity and/or quality of the oral microbiome, termed “oral dysbiosis“, have been associated with both common oral diseases such as periodontitis [2] and those that are less frequent but more serious, such as benign lesions [3], oral potentially malignant disorders (OPMDs) [4], and oral cancer [5].

Although benign oral lesions are not lethal, they can result in extensive loss of soft tissue and/or bone, and they are prone to recurrence [6]. Unfortunately, literature data related to their exact incidence are rare [6,7]. OPMDs are defined as oral mucosal lesions associated with a statistically increased risk of developing oral cancer [8]. Epidemiological studies have shown that almost 5% of the world population has OPMDs [9], while their overall malignant transformation rate is estimated at around 8% but can grow up to 40%, indicating the seriousness of the problem [10,11]. Oral cancer is the sixth most common malignancy worldwide, and more than 90% of these tumors are oral squamous cell carcinomas (OSCC) [12]. In 2020, over 350,000 new cases of oral cancer were diagnosed globally, resulting in more than 175,000 deaths. It is estimated that by 2040, the incidence of OSCC will increase up to 40%, with a corresponding increase in mortality [13].

Periodontitis is a highly prevalent oral inflammation, ranging from 20 to 50% across the world, triggered by bacteria [14]. The host’s immune response ultimately causes the breakdown of connective tissue and alveolar bone around the teeth [15]. There is a wealth of evidence linking chronic inflammation and the risk of neoplastic transformation, and some researchers point to the necessity of treating patients with periodontitis as a group at high risk of developing malignancy [5,16,17]. However, since the vast majority of people with periodontitis do not develop oral tumors, the link between these pathologies is not straightforward, and studies dealing with the identification and quantification of microorganisms in patients with oral tumors have not established a causal relationship between oral dysbiosis and oral cancer [18]. The presence of various periodontal pathogens, including *Prevotella melaninogenica* and *Prevotella intermedia*, and some oral commensals, such as *Streptococcus mitis*, has been investigated in patients with oral tumors and premalignant lesions, but the focus has been more on “who is present“ rather than “what they are up to“ [19]. The mere existence of certain bacteria in saliva, supra- or subgingival biofilm, and even in the tumor tissue itself does not automatically label them as the causal force of neoplastic transformation and progress, but may indicate just an opportunistic infection sustaining the conditions of the already altered oral mucosa. However, bacterial damage is for sure the culprit in the activation of inflammatory signaling cascades, which might in turn lead to malignant alteration [20]. It is therefore essential to show not only the bacterial presence in different types of altered oral mucosa, but simultaneous changes in chronic inflammation and neoplastic transformation markers as well. Key players in these processes are involved in cell cycle regulation, apoptosis, nutrient metabolism and/or protein synthesis, and tumor angiogenesis. PIK3K-AKT-mTOR signaling pathway modulation is described as one of the crucial mechanisms connecting chronic inflammatory conditions and malignancies via cellular growth stimulation [21]. The mitogen-activated protein kinase (MAPK) cascades are well-defined signaling modules, regulated by phosphatases (DUSP16, among others), and have been demonstrated to play important roles in a variety of cellular responses, such as proliferation, differentiation, and apoptosis [22]. Cyclooxygenase (COX) is an enzyme involved in the conversion of arachidonic acid to prostaglandin H2 and is implicated in cancerogenesis via cell proliferation, angiogenesis, and inhibition of apoptosis [23].

Therefore, the aims of the present study were as follows: (i) to determine the frequencies of *Prevotella intermedia*, *Prevotella melaninogenica*, and *Streptococcus mitis* in oral benign lesions, OPMDs, and OSCC tissues in periodontitis patients; and (ii) to analyze the expression patterns of possible candidate genes (*PI3CA/AKT2/mTOR*, *DUSP16/MAPK14*, *COX2*) that might be modulated by the presence of the aforementioned bacteria.

## 2. Materials and Methods

### 2.1. Participants and Study Design

This cross-sectional research study was conducted between January 2020 and December 2022. The study design was approved by the Ethical Committee of the School of Dental Medicine, University of Belgrade (No. 36/7) and was in accordance with the Declaration of Helsinki’s ethical and scientific principles.

The research included consecutive patients referred for examination, diagnosis, and possible treatment of suspicious oral mucosal lesions (benign lesions, OPMDs, or OSCCs) to the Department of Oral Medicine and Periodontology or to the Department of Maxillofacial Surgery, School of Dental Medicine, University of Belgrade. Patients were thoroughly informed about the study, its purpose, and potential risks, and they signed the informed consent prior to entering the study.

The inclusion criteria were the following: (1) adult patients aged ≥ 18 years; (2) presence of a suspicious oral mucosal lesion; (3) confirmed periodontal disease (periodontitis) with at least 10 teeth remaining; and (4) toothless patients with a known history of periodontal disease. The exclusion criteria were the following: (1) periodontal treatment coupled with the use of antibiotics, antiseptics, or anti-plaque mouthwashes within the last two months; (2) presence of dentures; and (3) history of radiotherapy and/or chemotherapy in the head and neck region. A total of ninety patients met the abovementioned criteria, and their detailed anamnestic data were collected and recorded in the predesigned medical records.

A comprehensive head and neck examination was conducted along with a visual assessment of the oral cavity during the clinical examination [24].

Periodontal parameters, such as periodontal probing depth (PPD), clinical attachment level (CAL), and gingival recession (GR), were measured at six points around every present tooth (mesio-buccal, mid-buccal, disto-buccal, mesio-lingual, mid-lingual, and disto-lingual) using a periodontal probe (North Carolina–Hu-Friedy, Chicago, IL, USA). Oral hygiene status was recorded using the bleeding on probing (BOP) index and plaque index (PI) in the same manner [25].

The periodontal stage of patients was determined using periodontal parameters such as tooth presence and X-ray-detected bone loss [26].

### 2.2. Sample Collection

The most suspicious parts of the OPMDs and OSCCs were observed using 2.5× magnification glasses (Keeler, Windsor, UK). Samples were collected by the incisional biopsy technique, while the collection of benign lesions was performed by means of an excisional biopsy. After local anesthesia administration, a surgical #11 blade was used to collect the tissue sample. All lesions were sampled according to the clinical experience of two clinicians (a specialist in oral medicine and a specialist in maxillofacial surgery). All the tissue samples were cut into two fragments; one was placed into sterile Eppendorf tubes containing RNAlater solution (Ambion, Thermo Fisher Scientific Inc., Waltham, MA, USA) and stored at −70 °C until RNA extraction. The second tissue fragment was immediately immersed in a 10% formalin solution (Merck, Darmstadt, Germany) and subsequently paraffin embedded for histopathological verification. Absorbable sutures were placed, and antibiotics (Amoxicilline 0.5 g, 3 times daily for 5 days) were prescribed.

Following histopathological verification, out of ninety patients, 25 were classified as benign lesions, 30 as OPMDs, and 35 as OSCCs.

### 2.3. DNA Extraction and Microorganisms’ Detection

Tissue samples were paraffin embedded and cut on a microtome (Leica RM2245, Nussloch, Germany) at 4 µm thickness. After xylene deparaffinization, standard phenol-chloroform DNA extraction was performed. TaqMan-based quantitative polymerase chain reaction (qPCR) was performed in order to detect the presence of microorganisms. The species-specific PrimeTime qPCR assays (Integrative DNA Technologies, Coralville, IA, USA) were designed, and the sequences of each primer-probe set are given in Table 1. The qPCR mix in the final volume of 15 µL contained 20 ng of a DNA template, 0.5 µM of each primer, 0.25 µM of each TaqMan probe, 1× FastGene^®^ Probe qPCR Universal Mix (NIPPON Genetics EUROPE, Germany), and sterilized nuclease-free water. The temperature profiles were the following: 10 min at 95 °C, followed by 45 cycles of 15 s at 95 °C and 30 s at 58 °C. For each sample, glyceraldehyde 3-phosphate dehydrogenase (GAPDH) was amplified in order to exclude false negative findings, and in each run, a positive control was run: a DNA sample extracted from strains acquired from the American Type Culture Collection (ATCC): *P. intermedia* (ATCC33563), *P. melaninogenica* (ATCC25845), and *Str. mitis* strain NCTC 12261 (ATCC49456).

### 2.4. RNA Extraction, Reverse Transcription, and Real-Time PCR Relative Gene Expression Analysis

TRIzol reagent (Thermo Fisher Scientific, Waltham, MA, USA) was used to extract total RNA from the samples in accordance with the manufacturer’s instructions. Prior to RNA extraction, tissue samples were homogenized in 1 mL of TRIzol reagent using a tissue disrupter (Sonopuls HD 2070/2200; Bendlin Electronics GmbH and Co. KG, Berlin, Germany). After the isolation, complementary DNA from total RNA (1 μg) was generated using the Transcriptme RNA Kit (Blirt SA, Gdansk, Poland). The FastGene ICGreen 2× PCR Universal Mix (NIPPON Genetics EUROPE, Germany) was used to amplify segments of *PIK3CA*, *DUSP16*, *AKT2*, *mTOR*, *MAPK14*, and *COX2* genes in the presence of the corresponding primers (Table 1). Glyceraldehyde 3-phosphate dehydrogenase (GAPDH) was used as an endogenous control for normalization (primer sequences are also listed in Table 1). The following conditions for the qPCR were used: a holding stage of 95 °C for 3 min and a cycling stage with 45 cycles of 95 °C for 30 s, followed by 60 °C for 30 s, and 72 °C for 30 s. Each qPCR run was performed using the CFX96 real-time system (Bio-Rad Laboratories, Hercules, CA, USA), followed by melting curve analysis in order to confirm the amplification specificity. All the samples were run in duplicate. The results were obtained as threshold cycle (Ct) values, and the relative expression levels were calculated using the ∆∆Ct method [27]. The mean value of duplicates for each sample was calculated, and the relative gene expression levels of the selected genes were defined as the ratio of each gene to the glyceraldehyde 3-phosphate dehydrogenase expression.

### 2.5. Statistical Analysis

The statistical analyses were performed using Statistical Package for Social Science (SPSS software package, version 22.0; SPSS Inc., Chicago, IL, USA) and GraphPad Prism 9.0.00 (GraphPad Software, San Diego, CA, USA). Categorical data were studied using Pearson’s χ^2^ test or Fisher’s exact test, depending on the sample size. Descriptive statistics were presented as mean ± standard deviation and median values. The distribution of continuous outcome values was examined using the Kolmogorov–Smirnov normality test. Owing to the deviation from the normal distribution and given that all three study groups differed in size, i.e., in the number of patients, non-parametric statistical tests were used. Specifically, the Kruskal–Wallis H-test was applied for comparisons between all three study groups and the Mann–Whitney U-test for comparisons between each of the two groups.

## 3. Results

### 3.1. Clinical and Epidemiological Findings

The benign lesions group consisted of fibromas, papillomas, frictional keratoses, and one benign granular cell tumor. OPMD lesions included leukoplakias, erythroplakias, erythro-leukoplakias, and sublingual keratoses. Out of all OSCCs, two were subcategorized as verrucous carcinomas and one as carcinoma in situ. Among OSCC patients, 7 (20%) were classified as clinical stage 1, 8 (22.9%) as clinical stage 2, 11 (31.4%) as clinical stage 3, and 9 (25.7%) as clinical stage 4. As for the histological grade, 10 (28.6%) carcinomas were grade 1, 23 (65.7%) were grade 2, and 2 (5.7%) were grade 3.

No statistically significant difference was observed between the benign, OPMD, and OSCC groups regarding the patients’ age (*p* = 0.322, Table 2). On the other hand, OPMD and OSCC lesions were more frequent in male patients, while benign lesions were predominant in female patients (*p* = 0.013, Table 2). The majority of patients with OSCC had the fourth stage of periodontal disease (19/31). More specifically, compared to all the other stages, including toothless patients, the presence of the fourth periodontal stage led to an almost four-fold increase in risk for the development of OSCC compared to benign lesions (Odds Ratio (OR) 3.76, 95% Confidence Interval (CI) 1.21–11.68, *p* = 0.019) and an almost five-fold increase in risk compared to OPMD (OR 4.75, 95%CI 1.56–14.48, *p* = 0.005). No significant difference was observed between patients with different types of lesions regarding the results of clinical periodontal measurements and the duration of the specific oral lesion (*p* > 0.05, Table 2).

### 3.2. Microbiological Findings

*Streptococcus mitis* was found very frequently in all three lesion types: 60% in benign lesions, 50% in OPMDs, and 69% in OSCC (Table 3), without a statistically significant difference between the groups (*p* > 0.05). *Prevotella melaninogenica* was the most prevalent of the three analyzed bacteria, with the frequency being 60% in benign lesions, 87% in OPMDs (*p* = 0.024), and 77% in OSCC. Logistic regression analysis shows that the presence of *P. melaninogenica* led to a 4.33-fold increase in the risk for the lesion to be potentially malignant (OR 4.33, 95% CI 1.15–16.26). *Prevotella intermedia* was the least frequent of the analyzed bacteria: 16%, 27%, and 31% in benign lesions, OPMD, and OSCC, respectively, and no significant difference was observed between the groups (*p* > 0.05). No significant difference was observed between the presence of bacteria and clinical stage or histological grade in OSCC patients.

### 3.3. Gene Expression Patterns

**Figure 1:**
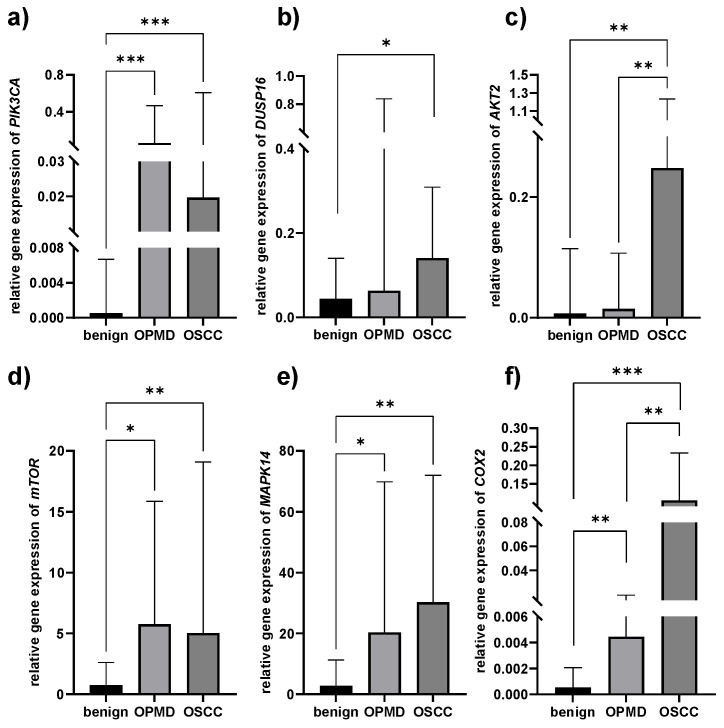
Relative gene expression levels of (**a**) PIK3CA, (**b**) DUSP16, (**c**) AKT2, (**d**) mTOR, (**e**) MAPK14, and (**f**) COX2 in benign lesions, oral potential malignant lesions (OPMDs), and oral squamous cell carcinomas (OSCCs). *—*p* > 0.005, **—*p* > 0.001, ***—*p* = 0.001.

A significantly higher relative gene expression level of PIK3CA was found both in OPMD and OSCC tissues compared to benign oral mucosal lesions (*p* = 0.001) (Figure 1a, Table 4). DUSP16 was only overexpressed in the OSCC group when compared to benign lesions (*p* = 0.05, Figure 1b, Table 4). AKT2 was significantly upregulated in OSCC tissues, compared both to benign and OPMD tissues (*p* = 0.002) (Figure 1c, Table 4). Similar to PIK3CA, mTOR was also overexpressed in OPMD and OSCC tissues, compared to benign (*p* = 0.016 and *p* = 0.002, respectively, Figure 1d, Table 4), as well as MAPK14 (*p* = 0.007 and *p* = 0.001, respectively, Figure 1e, Table 4). The COX2 expression level was the lowest in benign lesions and the highest in OSCC tissues (*p* = 0.003 for benign vs. OPMD, *p* = 0.004 for OPMD vs. OSCC, and *p* = 0.001 for benign vs. OSCC, Figure 1f, Table 4).

### 3.4. Correlation between Bacterial Presence and Gene Expression

**Figure 2:**
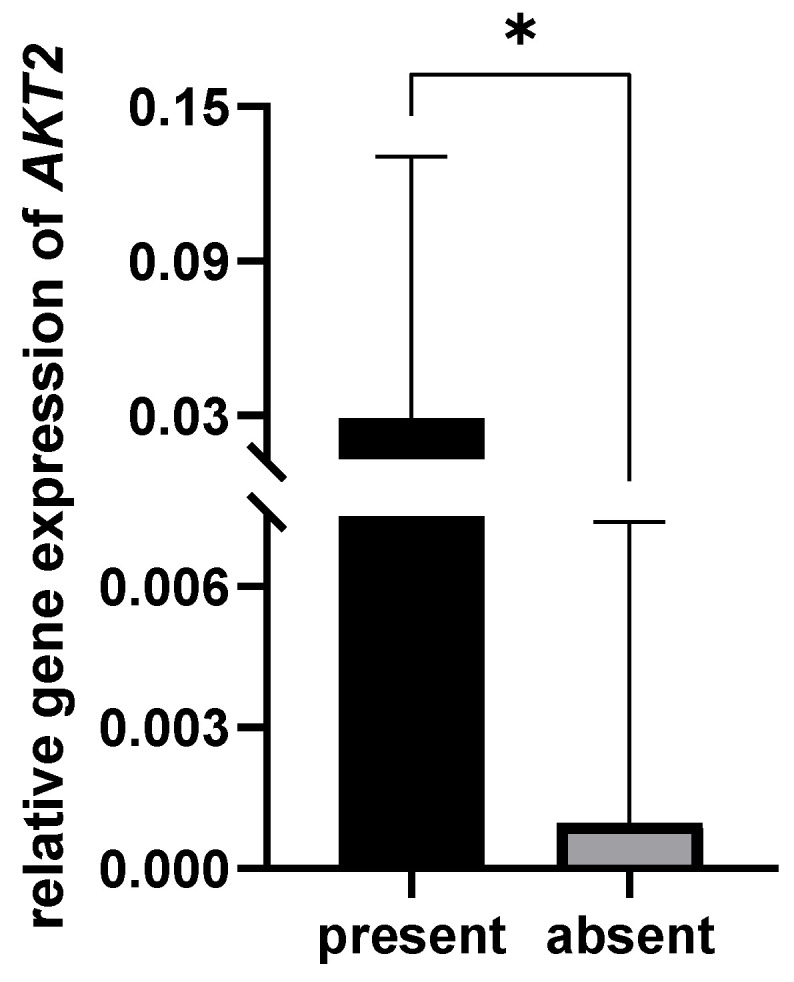
Relative gene expression of *AKT2* in OPMD lesions with and without *Prevotella melaninogenica.* * *p* < 0.05.

The OPMD tissues in which *Prevotella melaninogenica* was present exhibit a higher expression level of AKT2 (*p* = 0.042, Figure 2). Additionally, a positive correlation was found between the presence of *P. melaninogenica* and the relative expression of the *AKT2* gene (ρ = 0.374, *p* = 0.042) in the OPMD lesions.

**Figure 3:**
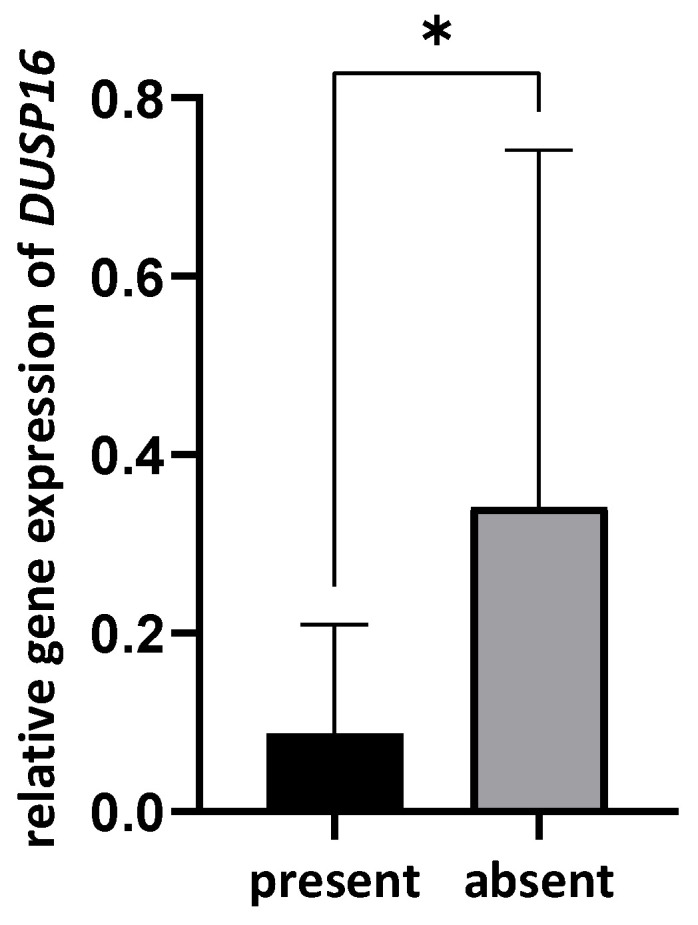
Relative gene expression of DUSP16 in OSCC tissues with and without *Streptococcus mitis.* * *p* < 0.05.

In contrast, significantly lower expression of DUSP16 was observed in OSCC tissues containing *Streptococcus mitis* (*p* = 0.011, Figure 3). A negative correlation was observed between the presence of *Str. mitis* and the relative expression of the *DUSP16* gene (ρ = −0.387, *p* = 0.029) in the OSCCs. No other correlations were established.

## 4. Discussion

The oral cavity is inhabited by a usually symbiotic, complex ecosystem consisting of numerous bacterial species, viruses, fungi, and other microorganisms [28,29]. However, qualitative and/or quantitative disruptions of the oral microbiome, the so-called “oral dysbiosis”, may occur, leading to chronic inflammation, a condition that has been implicated in the pathogenesis of multiple diseases, including oral cancer.

Periodontitis is a chronic inflammatory disease shown to be associated with OPMD and OSCC [4,17,30]. Komlós et al. found that most of the OSCC patients had stage IV periodontitis [31], and our study corroborates these findings since the majority of stage IV periodontitis patients included in the study had developed OSCC. Given that all the patients had periodontitis, it is understandable that no significant difference was observed between the three groups regarding clinical periodontal parameters, such as PPD and CAL. The results of the present study also show a male predominance of OSCC and OPMD lesions. This finding could be partially explained by their more frequent smoking and alcohol consumption habits [32], although there was no significant difference in these environmental factors between patients with different lesions.

Numerous studies have attempted to uncover microbial changes involved in oral cancer development and progression and reveal new potential therapeutic targets [20,33], but literature data remain inconclusive, especially in terms of the signaling pathways affected by the microorganisms. Therefore, one of the aims of the present study was to assess the frequency of the periopathogenic bacteria *Prevotella melaninogenica, Prevotella intermedia*, and *Streptococcus mitis* in different oral pathological conditions. *Prevotella* is a genus of Gram-negative bacteria frequently found in the oral cavity. Although undoubtedly linked to the development of periodontitis, the role of *Prevotella* species in oral carcinogenesis is still under debate. For instance, Zang and co-workers [34] showed that *Prevotella* species were more frequent in the tumor tissues of oral cancer patients than in the adjacent normal tissue. In our study, all three examined microorganisms were present at varying frequencies, the lowest being 16% for *P. intermedia* in benign lesions and the highest being 87% for *P. melaninogenica* in OPMD lesions. *P. melaninogenica* was the most frequent of the three bacteria, and a significant difference in its distribution between benign lesions and OPMD (*p* = 0.024) was established. In fact, the results of the logistic regression analysis show that the presence of *P. melaninogenica* leads to a 4.3-fold increase in the risk that the lesion will be potentially malignant. On the other hand, the other representative of the genus, *Prevotella*, *P. intermedia,* was scarcely present in oral mucosal lesions. This finding contradicts the study of Zhang and co-workers [30], who found a high prevalence of *P. intermedia* in the tumor tissues, suggesting a potential association with OSCC. On the other hand, Moghimi and coworkers [35] reported 29% of OSCC samples to be positive for the presence of *P. intermedia*, which is quite similar to the 31% found in the present study. The literature data show higher counts of *P. melaninogenica* and *S. mitis* in saliva samples of patients with OSCC compared to non-OSCC patients [36,37]. The study of Gopinath and co-workers [38] established that the bacteriome in tumor tissue and both paired swab and whole mouth fluid samples from oral cancer patients differed significantly in terms of overall community structure. Since the focus of our study was to evaluate the influence of the presence of bacteria in the mucosal lesion on gene expression changes, we opted for the deeper layers of the lesions, collected by means of tissue biopsy. Contrary to our findings, Robayo and coworkers did not detect *P. melaninogenica* in oropharyngeal carcinoma tissue samples [39], but de Martin et al. confirmed its presence in the tonsillar OSCC [40]. A recent study by Zheng et al. using in situ hybridization established the presence of *P. melaninogenica* in oral lichen planus [41].

Several possible mechanisms have been suggested in regard to the role of oral microbiota in cancer pathogenesis: (i) stimulation of local and/or systemic chronic inflammation, during which the inflammatory mediators initiate or facilitate cell proliferation and mutagenesis; (ii) the activation of NF-κB and apoptosis inhibition; or (iii) the production of some carcinogenic substances [42]. The present study aimed to analyze the gene expression patterns of some potential candidate genes that might be modulated by the presence of the abovementioned bacteria. The phosphoinositide 3-kinase (*PI3K*) *AKT/mTOR* signaling pathway is involved in protein synthesis, autophagy, cell cycle regulation, apoptosis, nuclear protein organization, and nutrient metabolism, and its upregulation is associated with the risk of OSCC formation [43,44,45,46,47]. The results of the present study support these findings, since *PIK3CA*, *AKT2*, and *mTOR* were overexpressed in OPMD and/or OSCC compared to benign lesions. The activation of the mitogen-activated protein kinase (MAPK) cascade transduces various extracellular signals to the nucleus, thus regulating gene expression, cell proliferation and differentiation, cell cycle arrest, and apoptosis. MAPKs’ activation is regulated by the dual-specificity protein phosphatases (DUSPs). More specifically, DUSP16 has been shown to interact with MAPK14 [48], which is involved in lymphangiogenesis, angiogenesis, and cell proliferation in head and neck cancer [22] and cancer cells growth control [49]. Additionally, it was found that *DUSP16* overexpression increases cell chemotherapy resistance and promotes cell proliferation [50,51]. Finally, the expression of *COX2* was also significantly higher in OPMD and OSCC compared to benign lesions, which is in line with the role of the encoded enzyme. Namely, *COX* is involved in the conversion of arachidonic acid to prostaglandin H2, an important precursor of prostacyclin, which is implicated in cancerogenesis via cell proliferation, angiogenesis, and inhibition of apoptosis [23].

The study by Zheng and co-workers [41] found that *P. melaninogenica* can adhere to the cell surface, invade macrophages, and increase IL-1β, IL-6, and TNF-α expression in oral lichen planus. The results of the present study show that *AKT2* was overexpressed in the presence of *P. melaninogenica* in OPMDs. Since *AKT2* gene activation is associated with OSCC pathogenesis [43], patients with OPMD lesions with *AKT2* overexpression should be closely monitored owing to the higher probability of malignant alteration.

There are different reports about the presence and effect of *Streptococcus mitis* in oral mucosal lesions, some of them suggesting cancerous and others anti-cancerous effects [20,52,53]. The protective (anti-cancer) effect of *Str. mitis* was attributed to hydrogen-peroxide production and to T-cell mediated immunity [54,55]. Our study demonstrates that *DUSP16* expression was downregulated in the presence of *S. mitis* in OSCC. Since we also found a higher *DUSP16* expression in OSCC compared to benign oral mucosal lesions, *Str. mitis* putatively exhibits a protective effect in OSCC via *DUSP16* downregulation.

*P. intermedia* is a Gram-negative anaerobic bacterium associated with different pathologies in the oral cavity, such as periodontal disease and oral squamous cell carcinoma [56]. Despite the strong literature evidence of *PI3K*, *MAPK*, and *COX2* activation by *P. intermedia* [57,58,59], our study did not confirm the microorganism’s influence on the investigated molecules and pathways.

## 5. Conclusions

The present study suggests a significant contribution of *P. melaninogenica* and *Str. mitis* in the pathogenesis of oral mucosal lesions. They might exhibit their corresponding roles via *AKT2* upregulation in OPMD and *DUSP16* downregulation in OSCC. Further investigations should be performed on larger cohorts to investigate the abundance of the analyzed bacteria and confirm the obtained results.

## Figures and Tables

**Table 1:** List of primers and probes used in this study.

Microorganism/Gene	Primer and/or Probe Sequence
*Prevotella intermedia*	Fwd: GACCCGAACGCAAAATACAT Rv: AGGGCGAAAAGAACGTTAGG Probe: FAM-AAAGAAGGAACACCCCGACT-TAMRA
*Prevotella melaninogenica*	Fwd: GTGGGATAACCTGCCGAAAG Rv: CCCATCCATTACCGATAAATCTTTA Probe: FAM-CAAATCTGATGCCGTCATCGAAGACTATGC-TAMRA
*Streptococcus mitis*	Fwd: TTTTGTCATCTAGCCTTGC Rv: GCAGTCATATCATCACCTTC Probe: FAM-ACTTGGGCAATCCCGACAGATTCTAAC-TAMRA
*GAPDH*	Fwd: GGGCTCTCCAGAACATCATCC Rv: GTCCACCACTGACACGTTGG Probe: FAM-CCTCTACTGGCGCTGCCAAGGCT-TAMRA
*PIK3CA*	Fwd: TTACCCTCTTCTGCCGGAGG Rv: AAGTGGATGCCCCACAGTTC
*DUSP16*	Fwd: AGGTGGGTTTGCTGAGTTCTC Rv: CTCGGGGATAAAGTCAGGCTT
*AKT2*	Fwd: GCAAAGCAGGAGTATAAGAAAGGAA Rv: GCAGAGAGGTAATCAGCACCAA
*mTOR*	Fwd: GCCGCGCGAATATTAAAGGAA Rv: TGGTTTCCTCATTCCGGCTC
*MAPK14*	Fwd: ACTGGCTCGGCACACAGATG Rv: TCCCACTGACCAAATATCAACTG
*COX-2*	Fwd: CAGCACTTCACGCATCAGTT Rv: CGCAGTTTACGCTGTCTAGC
*GAPDH*	Fwd: ATGGGGAAGGTGAAGGTCG Rv: GGGGTCATTGATGGCAACAATA

**Table 2:** Patient data obtained upon examination.

	Benign (n = 25)	OPMD (n = 30)	OSCC (n = 35)	*p* Value
age (mean ± SD; median)	58.29 ± 2.79; 58	54.29 ± 3.82; 50	60.39 ± 2.41; 63	0.322
sex (male/female)	6/19	18/12	20/15	**0.013**
smoking (yes/used to smoke/no)	5/9/11	8/15/7	8/14/13	0.596
alcohol (yes/used to drink/no)	17/3/5	17/4/9	23/5/7	0.871
periodontal stage (1/2/3/4/toothless)	0/11/7/6/1	2/10/9/6/3	0/6/7/19/3	**0.047**
periodontal probing depth (mm) (mean ± SD, median)	3.55 ± 0.15; 3.5	3.05 ± 0.22; 3.2	3.47 ± 0.22; 3.2	0.295
clinical attachment level (mm) (mean ± SD, median)	4.29 ± 0.30; 3.8	3.91 ± 0.36; 3.77	4.73 ± 0.49; 4.1	0.720
plaque index (mean ± SD, median)	0.60 ± 0.06; 0.51	0.63 ± 0.08; 0.76	0.69 ± 0.08; 0.78	0.327
bleeding on probing (mean ± SD, median)	0.62 ± 0.06; 0.54	0.63 ± 0.07; 0.65	0.72 ± 0.07; 0.8	0.243
duration of oral lesion (in months) (mean ± SD, median)	20.52 ± 6.72; 12	14.43 ± 5.6; 6	12.30 ± 5.23; 4	0.111

**Table 3:** The frequency of positive/negative findings for *Streptococcus mitis*, *Prevotella melaninogenica*, and *Prevotella intermedia* in benign lesions, OPMDs, and OSCC tissue samples.

Bacteria	Benign (n = 25)	OPMD (n = 30)	OSCC (n = 35)		*p* Value	OR (95%CI)
*Streptococcus mitis* (y/n)	15/10	15/15	24/11	benign vs. OPMD	0.458	0.67 (0.23–1.95)
				benign vs. OSCC	0.493	1.45 (0.50–4.25)
				OPMD vs. OSCC	0.128	2.18 (0.79–5.99)
*Prevotella melaninogenica* (y/n)	15/10	26/4	27/8	benign vs. OPMD	**0.024**	**4.33 (1.15–16.26)**
				benign vs. OSCC	0.153	2.25 (0.73–6.92)
				OPMD vs. OSCC	0.325	0.519 (0.14–1.94)
*Prevotella intermedia* (y/n)	4/21	8/22	11/24	benign vs. OPMD	0.340	1.91 (0.50–7.30)
				benign vs. OSCC	0.174	2.41 (0.67–8.70)
				OPMD vs. OSCC	0.671	1.26 (0.43–3.71)

**Table 4:** Median values with the corresponding range of relative gene expression levels for all the analyzed genes.

Gene Expression Median (Range)	Benign (n = 25)	OPMD (n = 30)	OSCC (n = 35)	Comparison	*p* Value
*PIK3CA*	0.0007 (0.00001–0.28)	0.05 (0.00005–7.98)	0.027 (0.00–57.65)	benign vs. OPMD	0.0001
				benign vs. OSCC	0.0001
				OPMD vs. OSCC	0.717
*DUSP16*	0.039 (0.0002–0.56)	0.06 (0.00013–20.98)	0.14 (0.02–2.48)	benign vs. OPMD	0.161
				benign vs. OSCC	0.050
				OPMD vs. OSCC	0.990
*AKT2*	0.009 (0.00002–0.66)	0.009 (0.00001–2.66)	0.25 (0.00005–15.61)	benign vs. OPMD	0.859
				benign vs. OSCC	0.002
				OPMD vs. OSCC	0.002
*mTOR*	0.72 (0.009–23.6)	4.43 (0.0022–171.65)	5.03 (0.0006–181.9)	benign vs. OPMD	0.016
				benign vs. OSCC	0.003
				OPMD vs. OSCC	0.664
*MAPK14*	2.89 (0.03–98.38)	19.38 (0.15–432)	30.21 (0.0055–223.12)	benign vs. OPMD	0.007
				benign vs. OSCC	0.001
				OPMD vs. OSCC	0.580
*COX2*	0.0007 (0.00001–0.66)	0.004 (0.00001–0.71)	0.091 (0.00003–1.03)	benign vs. OPMD	0.003
				benign vs. OSCC	0.0001
				OPMD vs. OSCC	0.004

## Data Availability

Not applicable.
